# Precision oncotherapy based on liquid biopsies in multidisciplinary treatment of unresectable recurrent rectal cancer: a retrospective cohort study

**DOI:** 10.1007/s00432-019-03046-3

**Published:** 2019-10-16

**Authors:** Stefano Guadagni, Giammaria Fiorentini, Michele De Simone, Francesco Masedu, Odisseas Zoras, Andrew Reay Mackay, Donatella Sarti, Ioannis Papasotiriou, Panagiotis Apostolou, Marco Catarci, Marco Clementi, Enrico Ricevuto, Gemma Bruera

**Affiliations:** 1 Department of Applied Clinical Sciences and Biotechnology, University of L’Aquila, 67100 L’Aquila, Italy; 2 Department of Oncology and Hematology, Azienda Ospedaliera “Ospedali Riuniti Marche Nord”, Pesaro, Italy; 3 Department of Surgical Oncology, Institute of Candiolo-IRCCS, Turin, Italy; 4 Department of Surgical Oncology, University of Crete, Heraklion, Greece; 5 Research Genetic Cancer Centre International GmbH, Zug, Switzerland; 6 Research Genetic Cancer Centre S.A, Florina, Greece; 7 General Surgery Unit, “C. e G. Mazzoni” Hospital, Ascoli Piceno, Italy; 8 Oncology Territorial Care S. Salvatore Hospital, Oncology Network ASL1 Abruzzo, L’Aquila, Italy

**Keywords:** Rectal cancer, Recurrence, Perfusion, Precision oncotherapy, Liquid biopsy

## Abstract

**Background:**

Third line innovative systemic treatments and loco-regional chemotherapy by hypoxic pelvic perfusion (HPP) have both been proposed for the treatment of unresectable not responsive recurrent rectal cancer (URRC). In the present study, we have compared the safety and efficacy of HPP/target therapy, using drug regimens selected by liquid biopsy precision oncotherapy, to third-line systemic therapy based on tissue specimens precision oncotherapy.

**Methods:**

HPP/target therapy regimens were selected based on precision oncotherapy, including assays for chemosensitivity and viability, and qRT-PCR for tumor-related gene expression. In the control group, systemic third-line and further lines of therapy were defined according to clinical and biological parameters.

**Results:**

From 2007 to 2019, 62 URRC patients were enrolled, comprised of 43 patients in the HPP/target-therapy group and 19 patients in the systemic therapy control group. No HPP related complications were reported and the most common adverse events were skin and bone marrow toxicity. In the HPP/target-therapy group, the ORR was 41.8% whereas in the systemic therapy control group was 15.8%. DCR of the HPP/target-therapy group was significantly improved over the systemic therapy group (*P* = 0.001), associated with a PFS of 8 vs 4 months (*P* = 0.009), and OS of 20 vs 8 months (*P* = 0.046).

**Conclusions:**

The present data indicate that in URCC patients, the integration of HPP/target-therapy and precision oncotherapy based upon liquid biopsy is as effective and efficacious as third-line treatment in local disease control and, therefore, deserves to be further assessed and compared to conventional systemic treatments in future prospective randomized trials.

## Introduction

Local rectal cancer recurrences have reduced over the last 20 years but the results of treatment remain unsatisfactory (Lee et al. 2017). In tertiary centers, approximately 50% of locally recurrent rectal cancers are considered eligible for multidisciplinary treatments including extensive “ultra-radical resection”, intraoperative and/or external irradiation, peri-operative systemic chemotherapy and targeted-therapy but population-based studies indicate that only 35% of patients undergo tumor resection (Lee et al. 2017; Westberg et al. 2019). Standard treatments for unresectable recurrent rectal cancer (URRC) include systemic chemo and radiotherapy but approximately 50% of patients do not respond (van Zoggel et al. 2018; Susko et al. 2016). Furthermore, 60% of patients in progression after first-line intensive systemic chemotherapy do not respond to second-line systemic therapy (Bruera et al. 2014). Currently, only Japanese guidelines for the treatment of colorectal cancer include locoregional therapy as a third-line therapy for these patients (Watanabe et al. 2018), and this is only offered in a few specialists centers (Bonvalot et al. 2012).

Hypoxic pelvic perfusion (HPP) is a locoregional complex and multidisciplinary procedure, performed by surgeons, oncologists, radiologists and perfusionists, during which the pelvic circulation is isolated by blocking blood flow in the aorta and inferior vena cava with balloon catheters, and at thigh-level with pneumatic cuffs (Bonvalot et al. 2012; Guadagni et al. 2006; Guadagni et al. 2007; Guadagni et al. 2001; Guadagni et al. 2017; Varker and Wanebo 2010). The rational for HPP is based upon the possibility to expose tumors to higher drug concentrations and enhance the cytotoxicity of some chemotherapeutic agents by introducing conditions of hypoxia (Guadagni et al. 2006; Guadagni et al. 2007). Non-homogeneous and non-comparable studies of HPP efficacy in URRC patients, unresponsive to systemic chemotherapy and/or radiotherapy, have reported median survival times, ranging from 10 to 20 months (Begossi et al. 2008; Murata et al. 2014). To improve the efficacy of locoregional HPP chemotherapy and post-HPP treatment, precision oncotherapy and chemosensitivity tests (Yoon and Kim 2014) employing tissue specimens are under investigation, in accordance with American Society of Clinical Oncology (ASCO) and European Society for Medical Oncology (ESMO) recommendations (Sepulveda et al. 2017). Liquid biopsy has also been validated and approved by the USA Food and Drug Administration (FDA), as a useful prognostic method in various cancers (Karachaliou et al. 2015). It remains to be determined, however, whether liquid biopsy will become the mainstay in oncology practice (Hench et al. 2018).

Here, we present a retrospective study of URRC, in progression after two lines of systemic chemotherapy and radiotherapy, that evaluates and compares safety and efficacy in URCC patients subjected to HPP and target therapy, using different drug regimens based on precision oncotherapy, to a URRC patient control group treated with third and subsequent line systemic therapy selected by tissue specimens precision oncotherapy.

## Methods

This retrospective URRC patient study was performed at the University of L’Aquila, L’Aquila, Italy, after approval from the Ethics committee of ASL n.1, Abruzzo, Italy; Chairperson: G. Piccioli; protocol number 10/CE/2018; date of approval: 19 July, 2018 (n.1419), and included patients with unresectable disease with a predictable course. All patients received complete information about their disease and the implications of the proposed palliative treatment, in accordance with the Declaration of Helsinki and ethical standards of the L’Aquila University committee on human experimentation, and written consent was obtained.

### Patients eligibility

Patient eligibility criteria were as follows: (i) histological diagnosis of adenocarcinoma of the rectum; (ii) diagnosis of unresectable recurrent rectal cancer, defined by pelvic side-wall involvement, and/or growth into the sciatic notch, and/or involvement of the first and/or second sacral vertebra, and/or encasement of the bladder or iliac vessels; (iii) an increase in recurrent tumor size for at least 3 months following radiation or systemic chemotherapy; (iv) a performance status of 0–3 based upon the Eastern Cooperative Oncology Group (ECOG) scale; (v) a leukocyte count > 2500 cells/mm^3^ and platelet count > 50,000 cells/mm^3^; (vi) a serum creatinine concentration of ≤ 1.2 mg/dl; (vii) absence of liver failure, deep venous thrombosis, severe atherosclerosis or coagulopathy, and (viii) disease progression after two lines of systemic chemotherapy.

### Patients characteristics

From 2007 to 2019, 62 URRC patients, with disease progression following two lines of systemic chemotherapy and radiotherapy, were enrolled in this study. The HPP/target therapy group was comprised of 43 patients treated with different drug regimens selected by liquid biopsy precision oncotherapy and a control group of 19 patients treated with conventional systemic therapy, according to fitness, age, performance status (PS), and comorbidity status. Patient selection for HPP/target or systemic therapy was decided by the medical oncologist and not by patient refusal or lack of eligibility criteria. Patient demographic and baseline data are displayed in Table 1 and recurrences were subdivided into three groups, using a modification of Yamada’s classification: (Yamada et al. 2001) (i) localized (including also cases with invasion of uterus, vagina, bladder, prostate, and seminal vesicles), (ii) sacral and (iii) lateral groups. Pain, tiredness, loss of appetite and severity burden was moderate to severe for all 62 patients, based upon the Eastern Cooperative Oncology Group (ECOG) classification (approximately 65% ECOG 3) (Bruera et al. 1991).

**Table 1:** Characteristics of the 62 URRC patients submitted to HPP/target-therapy or systemic therapy

	All patients (*n* = 62)	HPP/target-therapy cohort (*n* = 43)	Systemic therapy cohort (*n* = 19)	*P* value (test)
Gender				
Male	40	26	14	0.32, ns
Female	22	17	5	(Student *t*)
Age (years, median/IQR) at the 1st treatment of the 3rd line	62/56–68	60/55–67	65/58–68	0.07, ns, (Mann–Whitney)
Previous treatments of primary tumor				
Neo-adjuvant chemo/RT	8	6	2	0.71, ns, (Chi square)
Abdominoperineal resection	32	23	9	0.66, ns, (Chi square)
Low anterior resection	30	20	10	0.66, ns, (Chi square)
Adjuvant chemo/RT	26	19	7	0.59, ns, (Chi square)
Previous treatments of recurrence				
Systemic therapy	62	43	19	–
Chemotherapy	62	43	19	–
Fluorouracil	61	43	18	–
Oxaliplatin	61	43	18	–
Irinotecan	62	43	19	–
Capecitabine	10	7	3	1.00, ns, (Fisher exact)
Targeted-therapy	58	40	18	1.00, ns, (Fisher exact)
Cetuximab	18	12	6	0.77, ns, (Fisher exact)
Bevacizumab	52	34	18	0.15, ns, (Fisher exact)
RT	18	14	4	0.54, ns, (Fisher exact)
Surgery	15	13	2	0.12, ns, (Fisher exact)
Yamada’s modified classification (Yamada et al. 2001)				
Localized^a^	13	9	4	0.88, ns, (Chi square)
Sacral	37	25	12	
Lateral	12	9	3	
Other metastatic sites				
Not	25	19	6	0.35, ns, (Student’t)
Yes	37	24	13	
Eastern Cooperative Oncology Group (ECOG)				
1	10	7	3	0.88, ns, (Chi square)
2	12	9	3	
3	40	27	13	
Interval time from URRC diagnosis and the 1st treatment of the 3rd line (months, median/IQR)	14/12–19	15/13–18	14/9–35	0.44, ns, (Mann–Whitney)
Number of cycles of the 3rd line (mean/SD)	2.58/1.10	2.44/1.07	2.89/1.14	0.13, ns, (Student’t)

*Chemo/RT* systemic chemotherapy/radiotherapy, *IQR* interquartile range, *SD* standard deviation, *URRC* unresectable recurrent rectal cancer, *ns* not significant

^a^This group included cases with invasion of uterus, vagina, bladder, prostate, seminal vesicles

### HPP techniques

**Fig. 1:**
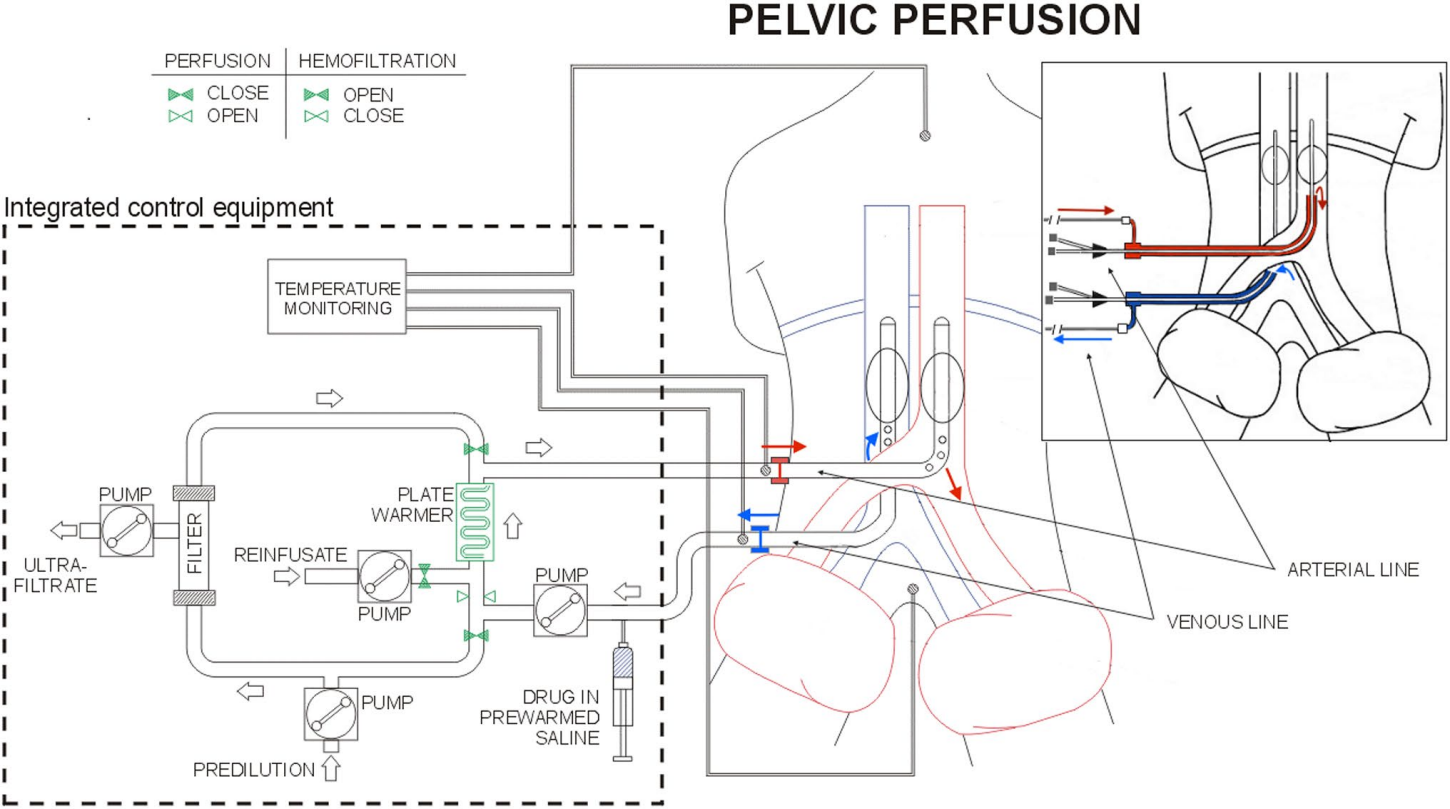
Schematic representation of hypoxic pelvic perfusion (HPP) with chemofiltration (surgical and percutaneous procedures)

Surgical and percutaneous HPPs were performed as previously described (Fig. 1) (Guadagni et al. 2017). Surgical approach was preferred for all patients and percutaneous for patients submitted to more than three perfusions. During femoral vessels surgical preparation, the venous balloon catheter was introduced in the femoral vein via saphenous vein to reduce the risk of femoral vein thrombosis. In patients exhibiting femoral vessel fibrosis, requiring two or three repeated perfusions, the surgical approach was achieved by exposing iliac vessels. Percutaneous perfusion was not performed if the diameter of the common femoral artery was ≤ 7 mm, making vessel dissection risky.

### Liquid biopsy, chemoresponse assay, and tumor gene expression

For each patient, blood samples (≈ 20 ml) were collected in sterile 50 ml Falcon tubes (4440100, Orange Scientific, Braine-l’Alleud, Belgium), containing 7 ml of 0.02 M EDTA anticoagulant (E0511.0250, Duchefa Biochemie B.V., Haarlem, The Netherlands), and stored at 2–8 °C. To ensure the stability and viability of circulating tumor cells (CTCs), tubes were stored on ice in impact-resistant transportation containers (Apostolou et al. 2017).

For sample preparation, whole-blood was layered over 4 ml polysucrose solution (Biocoll separating solution 1077, Biochrom, Berlin, Germany) and centrifuged for 20 min at 2500×*g*. Mononuclear cells, lymphocytes, platelets and granulocytes were collected after centrifugation and washed with phosphate-buffered saline (PBS, P3813; Sigma-Aldrich, Germany). Cells were incubated for 10 min in lysis buffer, comprised of 154 mM NH_4_Cl (31107; Sigma-Aldrich), 10 mM KHCO_3_ (4854; Merck, Germany) and 0.1 mM EDTA in deionized water, to lyse erythrocytes. Samples were centrifuged, washed in PBS and incubated with CD45-conjugated magnetic beads (39-CD45-250; Gentaur, Belgium), and pan-cytokeratin (pan-CK)-conjugated microbeads (MA1081-M; Gentaur), at 4 °C for 30 min. Following incubation, cells were collected in a magnetic field and washed in PBS. Purified CD45-negative/pan-cytokeratin positive cells were cultured in 12-well plates (4430400N; Orange Scientific) in RPMI-1640 plus 10% FBS for chemosensitivity, viability and qRT-PCR assays. Peripheral blood mononuclear cells (PBMCs) were purified and used as non-cancer cell controls. Circulating tumor cells (CTCs) were validated by RT-PCR, using specific primers for CK19 and panCK, and other cell types excluded using primers for CD31 and *N*-cadherin. Samples for chemosensitivity and gene expression assays contained ≥ 5 viable circulating tumor cells/ml.

For chemosensitivity assays (Apostolou et al. 2013), cells cultured in 12-well plates (3513, Corning) were treated with the following drug concentrations: 1 μM melphalan (Μ2011, Sigma-Aldrich), 1 μM doxorubicin (D1515, Sigma-Aldrich), 1 μM cisplatin (P4394, Sigma-Aldrich), 10 μM 5-fluorouracil (F6627, Sigma-Aldrich), 3 μM oxaliplatin (O9512, Sigma-Aldrich), 1 μM carboplatin (41575-94-4, Sigma-Aldrich), 5 μM irinotecan (I1406, Sigma-Aldrich), 1 μM raltitrexed (112887-68-0, Sigma-Aldrich) and 2 μM mitomycin C (M4287, Sigma-Aldrich). Cell viability was assessed by flow cytometry at 24-h intervals, for 6 days, using Annexin V-PE (559763; BD Bioscience). Living, dead and apoptotic cells were identified by flow cytometry (BD Instruments Inc., San José, CA, USA), using BD CellQuest Software (BD Instruments Inc). Validation of cell viability was also assessed using methyl-tetrazolium dye (MTT), crystal violet dye (CVE) and Sulfo-Rodhamine B (SRB) assays. The percentage of non-viable cancer cells was calculated under non-drug and drug-treated conditions, and chemosensitivity classified as: (1) non sensitivity < 35%; (2) partial sensitivity 35–80%, and (3) high sensitivity > 80%.

Gene expression was assessed by QRT-PCR (Apostolou et al. 2019). RNAs were purified from cultured cells, using the RNeasy Mini Kit (74105, Qiagen, Hilden, Germany) and 1 µg reverse transcribed using a PrimeScript RT Reagent Kit (RR037A, Takara, Beijing, China). Real-time qPCR, with KAPA SYBR Fast Master Mix (2×) Universal (KK4618, KAPA Biosystems, MA, USA), was performed in a final volume of 20 μl, using specific primers for epidermal growth factor receptor (EGFR), vascular endothelial growth factor receptor (VEGFR), Kirsten rat sarcoma virus (*KRAS*), neuroblastoma *RAS* viral oncogene homolog (*NRAS*), v-Raf murine sarcoma viral oncogene homolog B gene (*BRAF*) and reference gene 18SrRNA, designed using Beacon Designer 8, and for multidrug resistance gene-ABCB1 gene (MDR1), thymidylate synthase (TYMS), dihydrofolate reductase (DHFR), serine hydroxymethyltransferase 1 (SHMT1), DNA excision repair protein (ERCC1), glutathione S-transferases (GST), using Genamics Expression 1.1 software (Genamics, Hamilton, New Zealand). All primers were evaluated by BLAST for specificity. Following denaturation at 95 °C for 2 min, cDNAs were subjected to 45 PCR cycles, consisting of denaturation at 95 °C for 10 s, annealing at 59 °C for 30 s and elongation at 72 °C for 60 s. Melting-curve analysis was performed from 70 to 90 °C, with 0.5 °C increments for 5 s, at each step. Template-free and negative controls were used in all experiments. All reactions were performed in triplicate and analyzed by Livak relative quantification (Livak and Schmittgen 2001). Gene expression was also performed using normal PBMCs from each patient, as controls in Livak analyses, and quantified using the following equations:

$$\Delta {\text{Ct}}_{{({\text{threshold}}\,{\text{Cycle}})}} = {\text{Ct}}_{\text{target}} - {\text{Ct}}_{{18{\text{SrRNA}}}} ,$$

$$\Delta \Delta {\text{Ct}} = \Delta {\text{Ct}}_{{({\text{patient}}\,{\text{CTCs}})}} - \Delta {\text{Ct}}_({_\text{non-cancer cells}}_),$$

$${\text{Relative}}\,{\text{expression}}\,{\text{level}} = 2^{{ - \Delta \Delta {\text{Ct}}}} ,$$

$$\% \,{\text{Gene}}\,{\text{expression}} = 100 \times \left( {2^{{ - \Delta \Delta {\text{Ct}}}} - 1} \right).$$

Comparative % gene expression in CTCs and PBMCs was classified as: (1) < 50% low over-expression; (2) > 50% high over-expression. A detailed description of the gene expression panel analyzed in this study was more recently reported (Apostolou et al. 2019).

### Drug regimens

In the HPP/target-therapy cohort, drug regimens were chosen according to the following criteria: (i) mono-chemotherapy for CTCs with high sensitivity for one or more drug, with highest chemosensitivity indicating which drug; (ii) poly-chemotherapy was chosen for CTCs with partial sensitivity; (iii) for target-therapy, drug selection was based on high over-expression in comparative CTC/PBMC assays, with the highest percentage used to select each drug. Specifically, in HPP/target-therapy cohort, tumor gene expression analysis suggested the use of cetuximab in case of EGFR expression or bevacizumab in case of VEGFR expression. One of these target-therapy drugs was administered when comparative % gene expression between CTCs and PBMCs was > 50%, chosen based on the highest overexpression level. Individual tailored chemotherapy and target-therapy regimens administered to the 43 patient HPP/target-therapy cohort are reported in Table 2, and drug concentrations were chosen from previous phase I and II studies of locoregional chemotherapy for advanced colorectal cancer (Guadagni et al. 2018; Iaffaioli et al. 2006; Fiorentini et al. 2005).

**Table 2:** Precision oncotherapy tests of the 43 patients submitted for HPP, tailored chemotherapy and post-HPP targeted-therapy

Pt	IV-CTCs (/ml)	5-FU	Oxaliplatin	Irinotecan	Mitomycin	Doxorubicin	Cisplatin	Alkeran	Carboplatin	Raltitrexed	TC
Part A											
1	14.8	*S = *70%	*S = *75%	*S = *70%	*S = *82%	*S = *19%	*S = *70%	*S = *25%	*S = *70%	*S = *60%	MMC (25 mg/m^2^)
2	8.9	*S = *65%	*S = *70%	*S = *82%	*S = *80%	*S = *24%	*S = *65%	*S = *24%	*S = *80%	*S = *70%	MMC (25 mg/m^2^) IRI (100 mg/m^2^)
3	15.3	*S = *65%	*S = *70%	*S = *60%	*S = *80%	*S = *22%	*S = *65%	*S = *23%	*S = *60%	*S = *45%	MMC (25 mg/m^2^) OX (80 mg/m^2^)
4	12.2	*S = *48%	*S = *61%	*S = *48%	*S = *82%	*S = *23%	*S = *70%	*S = *23%	*S = *47%	*S = *28%	MMC (25 mg/m2)
5	16.2	*S = *70%	*S = *72%	*S = *42%	*S = *45%	*S = *20%	*S = *62%	*S = *56%	*S = *57%	*S = *60%	5-FU (1000 mg/m^2^) OX (80 mg/m^2^)
6	7.5	*S = *62%	*S = *75%	*S = *80%	*S = *58%	*S = *22%	*S = *55%	*S = *20%	*S = *70%	*S = *38%	OX (80 mg/m^2^) IRI (100 mg/m^2^)
7	9	*S = *53%	*S = *55%	*S = *35%	*S = *85%	*S = *35%	*S = *55%	*S = *23%	*S = *45%	*S = *20%	MMC (25 mg/m^2^)
8	15.1	*S = *55%	*S = *83%	*S = *60%	*S = *20%	*S = *25%	*S = *60%	*S = *20%	*S = *62%	*S = *50%	OX (80 mg/m^2^)
9	8.3	*S = *55%	*S = *60%	*S = *45%	*S = *80%	*S = *24%	*S = *50%	*S = *40%	*S = *50%	*S = *20%	MMC (25 mg/m^2^)
10	8.4	*S = *40%	*S = *85%	*S = *50%	*S = *20%	*S = *20%	*S = *50%	*S = *22%	*S = *55%	*S = *55%	OX (80 mg/m^2^)
11	8.3	*S = *50%	*S = *80%	*S = *65%	*S = *60%	*S = *40%	*S = *60%	*S = *20%	*S = *55%	*S = *20%	OX (80 mg/m^2^)
12	8.4	*S = *60%	*S = *65%	*S = *60%	*S = *81%	*S = *24%	*S = *65%	*S = *24%	*S = *65%	*S = *60%	MMC (25 mg/m^2^)
13	15.2	*S = *60%	*S = *60%	*S = *40%	*S = *85%	*S = *20%	*S = *60%	*S = *45%	*S = *50%	*S = *20%	MMC (25 mg/m^2^)
14	9.8	*S = *38%	*S = *35%	*S = *41%	*S = *91%	*S = *30%	*S = *60%	*S = *49%	*S = *50%	*S = *10%	MMC (25 mg/m^2^)
15	6.9	*S = *55%	*S = *65%	*S = *40%	*S = *85%	*S = *24%	*S = *55%	*S = *24%	*S = *60%	*S = *25%	MMC (25 mg/m^2^)
16	9.7	*S = *60%	*S = *62%	*S = *65%	*S = *83%	*S = *65%	*S = *68%	*S = *22%	*S = *65%	*S = *60%	MMC (25 mg/m^2^)
17	6.9	*S = *35%	*S = *42%	*S = *50%	*S = *80%	*S = *35%	*S = *55%	*S = *65%	*S = *48%	*S = *40%	MMC (25 mg/m^2^)
18	9.8	*S = *60%	*S = *62%	*S = *42%	*S = *75%	*S = *23%	*S = *60%	*S = *65%	*S = *60%	*S = *28%	MMC (25 mg/m^2^)
19	8.4	*S = *50%	*S = *75%	*S = *50%	*S = *80%	*S = *20%	*S = *45%	*S = *35%	*S = *60%	*S = *70%	MMC (25 mg/m^2^) OX (80 mg/m^2^) RAL (3 mg/m^2^)
20	12.2	*S = *58%	*S = *62%	*S = *40%	*S = *70%	*S = *20%	*S = *70%	*S = *25%	*S = *45%	*S = *70%	MMC (25 mg/m^2^) CIS (70 mg/m^2^) RAL (3 mg/m^2^)
21	8.3	*S = *25%	*S = *80%	*S = *70%	*S = *80%	*S = *24%	*S = *40%	*S = *20%	*S = *42%	*S = *20%	MMC (25 mg/m^2^) OX (80 mg/m^2^) IRI (100 mg/m^2^)
22	7.5	*S = *50%	*S = *50%	*S = *50%	*S = *80%	*S = *25%	*S = *65%	*S = *60%	*S = *55%	*S = *70%	MMC (25 mg/m^2^) RAL (3 mg/m^2^)
23	6.9	*S = *25%	*S = *55%	*S = *45%	*S = *80%	*S = *30%	*S = *50%	*S = *65%	*S = *45%	*S = *75%	MMC (25 mg/m^2^) RAL (3 mg/m^2^)
24	15.1	*S = *35%	*S = *50%	*S = *50%	*S = *50%	*S = *35%	*S = *45%	*S = *70%	*S = *75%	*S = *50%	ALK (30 mg/m^2^) CAR (100 mg/m^2^)
25	16.3	*S = *40%	*S = *55%	*S = *50%	*S = *60%	*S = *30%	*S = *45%	*S = *75%	*S = *75%	*S = *25%	ALK (30 mg/m^2^) CAR (100 mg/m^2^)
26	8.9	*S = *45%	*S = *70%	*S = *20%	*S = *25%	*S = *20%	*S = *60%	*S = *30%	*S = *60%	*S = *10%	OX (80 mg/m^2^)
27	16.2	*S = *31%	*S = *65%	*S = *20%	*S = *52%	*S = *72%	*S = *41%	*S = *25%	*S = *55%	*S = *20%	DOX (35 mg/m^2^) OX (80 mg/m^2^)
28	9.4	*S = *40%	*S = *45%	*S = *50%	*S = *70%	*S = *22%	*S = *45%	*S = *24%	*S = *65%	*S = *70%	MMC (25 mg/m^2^) RAL (3 mg/m^2^)
29	9.8	*S = *50%	*S = *50%	*S = *45%	*S = *70%	*S = *24%	*S = *40%	*S = *60%	*S = *50%	*S = *70%	MMC (25 mg/m^2^) RAL (3 mg/m^2^)
30	10	*S = *45%	*S = *70%	*S = *45%	*S = *75%	*S = *20%	*S = *60%	*S = *50%	*S = *45%	*S = *30%	OX (80 mg/m^2^) MMC (25 mg/m^2^)
31	9.5	*S = *40%	*S = *70%	*S = *48%	*S = *70%	*S = *35%	*S = *55%	*S = *45%	*S = *55%	*S = *20%	OX (80 mg/m^2^) MMC (25 mg/m^2^)
32	8.9	*S = *50%	*S = *75%	*S = *50%	*S = *75%	*S = *24%	*S = *60%	*S = *25%	*S = *60%	*S = *30%	OX (80 mg/m^2^) MMC (25 mg/m^2^)
33	16.2	*S = *45%	*S = *60%	*S = *45%	*S = *80%	*S = *35%	*S = *60%	*S = *30%	*S = *65%	*S = *25%	MMC (25 mg/m^2^)
34	7.5	*S = *60%	*S = *50%	*S = *45%	*S = *80%	*S = *20%	*S = *65%	*S = *45%	*S = *60%	*S = *10%	MMC (25 mg/m^2^)
35	8.9	*S = *50%	*S = *60%	*S = *35%	*S = *82%	*S = *35%	*S = *40%	*S = *20%	*S = *55%	*S = *30%	MMC (25 mg/m^2^)
36	6.9	*S = *60%	*S = *60%	*S = *50%	*S = *75%	*S = *20%	*S = *55%	*S = *25%	*S = *45%	*S = *10%	MMC (25 mg/m^2^)
37	16.3	*S = *50%	*S = *55%	*S = *20%	*S = *82%	*S = *35%	*S = *50%	*S = *30%	*S = *47%	*S = *30%	MMC (25 mg/m^2^)
38	8.9	*S = *65%	*S = *45%	*S = *20%	*S = *80%	*S = *24%	*S = *45%	*S = *20%	*S = *60%	*S = *20%	MMC (25 mg/m^2^)
39	14.8	*S = *50%	*S = *55%	*S = *30%	*S = *75%	*S = *35%	*S = *50%	*S = *20%	*S = *50%	*S = *30%	MMC (25 mg/m^2^)
40	6.9	*S = *40%	*S = *65%	*S = *25%	*S = *80	*S = *40%	*S = *35%	*S = *25%	*S = *40%	*S = *22%	MMC (25 mg/m^2^)
41	9.4	*S = *50%	*S = *50%	*S = *35%	*S = *85%	*S = *20%	*S = *41%	*S = *45%	*S = *50%	*S = *30%	MMC (25 mg/m^2^)
42	16.2	*S = *50%	*S = *60%	*S = *35%	*S = *80%	*S = *20%	*S = *50%	*S = *23%	*S = *60%	*S = *30%	MMC (25 mg/m^2^)
43	9.8	*S = *55%	*S = *55%	*S = *20%	*S = *82%	*S = *24%	*S = *40%	*S = *30%	*S = *60%	*S = *25%	MMC (25 mg/m^2^)

Pt	EGFR	VEGFR	*KRAS*	*NRAS*	*BRAF*	MDR1 (%)	TYMS (%)	DHFR (%)	SHMT1 (%)	ERCC1 (%)	GST (%)	TT
Part-B												
1	*E = *45%	*E = *35%	NM	NM	NM	*E* = 65%	*E* = 0%	*E* = 0%	*E* = 0%	*E *= 15%	*E* = 20%	
2	*E = *45%	*E = *45%	NM	NM	NM	*E* = 60%	*E* = 10%	*E* = 0%	*E* = 0%	*E* = 20%	*E* = 20%	
3	*E = *35%	*E = *45%	NM	NM	NM	*E* = 55%	*E* = 0%	*E* = 0%	*E* = 0%	*E* = 25%	*E* = 20%	
4	*E = *45%	*E = *70%	NM	NM	NM	*E* = 80%	*E* = 0%	*E *= 0%	*E* = 0%	*E* = 0%	*E* = 18%	BEV (5 mg/kg)
5	*E = *45%	*E = *70%	NM	NM	NM	*E* = 80%	*E* = 0%	*E *= 0%	*E* = 0%	*E* = 0%	*E* = 10%	BEV (5 mg/kg)
6	*E = *60%	*E = *85%	NM	NM	NM	*E* = 70%	*E* = 0%	*E* = 0%	*E* = 0%	*E *= 0%	*E* = 10%	BEV (5 mg/kg)
7	*E = *60%	*E = *55%	NM	NM	NM	*E* = 60%	*E* = 0%	*E* = 0%	*E* = 0%	*E* = 0%	*E* = 10%	CET (250 mg/m^2^)
8	*E = *10%	*E = *45%	NM	NM	NM	*E* = 65%	*E* = 0%	*E* = 0%	*E* = 0%	*E* = 0%	*E* = 10%	
9	*E = *20%	*E = *20%	NM	NM	NM	*E* = 70%	*E* = 10%	*E* = 0%	*E* = 0%	*E* = 0%	*E* = 10%	
10	*E = *70%	*E = *45%	NM	NM	NM	*E* = 58%	*E* = 0%	*E* = 0%	*E* = 0%	*E* = 0%	*E* = 12%	CET (250 mg/m^2^)
11	*E = *40%	*E = *45%	NM	NM	NM	*E* = 70%	*E* = 0%	*E* = 0%	*E *= 0%	*E* = 0%	*E* = 10%	
12	*E = *45%	*E = *45%	NM	NM	NM	*E* = 65%	*E* = 0%	*E* = 0%	*E* = 0%	*E* = 5%	*E* = 25%	
13	*E = *20%	*E = *30%	NM	NM	NM	*E* = 70%	*E* = 5%	*E* = 0%	*E *= 0%	*E* = 0%	*E* = 10%	
14	*E = *20%	*E = *20%	MT^12e2^	NM	NM	*E* = 75%	*E* = 15%	*E* = 0%	*E* = 0%	*E *= 0%	*E* = 25%	
15	*E = *30%	*E = *30%	NM	NM	NM	*E* = 80%	*E* = 0%	*E* = 0%	*E* = 0%	*E* = 0%	*E* = 10%	
16	*E = *10%	*E = *65%	NM	NM	NM	*E *= 65%	*E* = 0%	*E* = 0%	*E* = 0%	*E* = 0%	*E* = 20%	BEV (5 mg/kg)
17	*E = *50%	*E = *80%	MM	NM	NM	*E* = 70%	*E* = 0%	*E* = 0%	*E* = 0%	*E* = 0%	*E* = 10%	BEV (5 mg/kg)
18	*E = *60%	*E = *70%	NM	NM	NM	*E* = 70%	*E* = 0%	*E* = 0%	*E* = 0%	*E *= 0%	*E* = 10%	BEV (5 mg/kg)
19	*E = *50%	*E = *65%	NM	NM	NM	*E *= 65%	*E* = 0%	*E* = 0%	*E* = 0%	*E* = 0%	*E* = 10%	BEV (5 mg/kg)
20	*E = *50%	*E = *20%	NM	NM	NM	*E* = 55%	*E* = 0%	*E* = 0%	*E* = 0%	*E* = 0%	*E* = 10%	CET (250 mg/m^2^)
21	*E = *25%	*E = *40%	MT^12e2^	NM	NM	*E* = 80%	*E* = 10%	*E* = 0%	*E* = 0%	*E* = 20%	*E* = 20%	
22	*E = *65%	*E = *50%	NM	NM	NM	*E* = 70%	*E* = 0%	*E* = 0%	*E* = 0%	*E *= 0%	*E* = 5%	CET (250 mg/m^2^)
23	*E = *50%	*E = *80%	NM	NM	NM	*E* = 70%	*E* = 0%	*E* = 0%	*E* = 0%	*E* = 0%	*E* = 10%	BEV (5 mg/kg)
24	*E = *50%	*E = *20%	NM	NM	NM	*E* = 80%	*E *= 10%	*E* = 0%	*E* = 0%	*E* = 25%	*E* = 20%	CET (250 mg/m^2^)
25	*E = *50%	*E = *20%	NM	NM	NM	*E* = 60%	*E* = 0%	*E* = 0%	*E* = 0%	*E* = 0%	*E* = 10%	CET (250 mg/m^2^)
26	*E = *50%	*E = *30%	NM	NM	NM	*E* = 65%	*E* = 10%	*E* = 0%	*E* = 0%	*E *= 0%	*E* = 10%	CET (250 mg/m^2^)
27	*E = *80%	*E = *35%	NM	NM	NM	*E* = 60%	*E* = 0%	*E* = 25%	*E* = 0%	*E* = 0%	*E* = 0%	CET (250 mg/m^2^)
28	*E = *40%	*E = *35%	MT^12e2^	NM	NM	*E* = 65%	*E* = 5%	*E* = 25%	*E* = 10%	*E* = 0%	*E* = 15%	
29	*E = *60%	*E = *70%	NM	NM	NM	*E* = 70%	*E* = 0%	*E* = 0%	*E* = 0%	*E* = 0%	*E* = 0%	BEV (5 mg/kg)
30	*E = *70%	*E = *60%	NM	NM	NM	*E* = 70%	*E* = 0%	*E* = 0%	*E* = 0%	*E *= 0%	*E* = 0%	CET (250 mg/m^2^)
31	*E = *60%	*E = *55%	NM	NM	NM	*E* = 65%	*E* = 5%	*E* = 0%	*E* = 0%	*E *= 0%	*E* = 10%	CET (250 mg/m^2^)
32	*E = *60%	*E = *55%	NM	NM	NM	*E* = 65%	*E* = 5%	*E* = 0%	*E* = 0%	*E* = 0%	*E* = 15%	CET (250 mg/m^2^)
33	*E = *40%	*E = *30%	NM	MT^61e3^	NM	*E* = 70%	*E* = 0%	*E* = 0%	*E *= 0%	*E* = 25%	*E* = 20%	
34	*E = *40%	*E = *70%	NM	NM	NM	*E* = 80%	*E* = 5%	*E* = 10%	*E* = 0%	*E* = 0%	*E* = 10%	BEV (5 mg/kg)
35	*E = *50%	*E = *70%	NM	NM	NM	*E* = 70%	*E* = 0%	*E *= 0%	*E* = 0%	*E* = 0%	*E* = 10%	BEV (5 mg/kg)
36	*E = *50%	*E = *20%	NM	NM	NM	*E* = 65%	*E* = 0%	*E* = 0%	*E* = 0%	*E* = 0%	*E* = 15%	CET (250 mg/m^2^)
37	*E = *40%	*E = *30%	MT^13e2^	NM	NM	*E* = 60%	*E* = 5%	*E* = 0%	*E* = 0%	*E* = 0%	*E* = 10%	
38	*E = *40%	*E = *80%	NM	NM	NM	*E* = 70%	*E* = 5%	*E* = 0%	*E* = 0%	*E* = 15%	*E* = 15%	BEV (5 mg/kg)
39	*E = *25%	*E = *20%	NM	NM	NM	*E* = 70%	*E* = 10%	*E* = 0%	*E* = 0%	*E* = 0%	*E* = 10%	
40	*E = *40%	*E = *60%	NM	NM	NM	*E* = 60%	*E* = 0%	*E* = 0%	*E* = 0%	*E* = 0%	*E* = 10%	BEV (5 mg/kg)
41	*E = *50%	*E = *70%	NM	NM	NM	*E* = 70%	*E* = 10%	*E* = 0%	*E* = 0%	*E* = 0%	*E* = 10%	BEV (5 mg/kg)
42	*E = *40%	*E = *50%	NM	NM	NM	*E* = 60%	*E* = 0%	*E* = 10%	*E* = 0%	*E* = 0%	*E* = 10%	BEV (5 mg/kg)
43	*E = *40%	*E = *40%	MT^12e2^	NM	NM	*E* = 65%	*E* = 0%	*E* = 0%	*E* = 0%	*E* = 0%	*E* = 5%	

*Pt* patient, *IV-CTCS* isolated viable circulating tumor cells, *5-FU* 5 fluorouracil, *MMC* mitomycin, *IRI* irinotecan, *OX* oxaliplatin, *RAL* raltitrexed, *CAR* carboplatin, *DOX* doxorubicin, *ALK* alkeran, *TC* tailored chemotherapy, *TT* target-therapy, *S* sensitivity, *E* gene expression, *NM* no mutations, *MT* mutated type, *12e2* codon 12 of exon 2, *61e3* codon 61 of exon 3, *CET* cetuximab, *BEV* bevacizumab, *EGFR* epidermal growth factor receptor, *VEGFR* vascular endothelial growth factor receptor, *KRAS* Kirsten rat sarcoma virus, *NRAS* neuroblastoma RAS viral oncogene homolog, *BRAF* v-Raf murine sarcoma viral oncogene homolog B gene, *MDR1* multidrug resistance gene (ABCB1 gene), *TYMS* thymidylate synthase gene, *DHFR* dihydrofolate reductase, *ERCC1* DNA excision repair protein, *GST* glutathione *S*-transferases

Systemic therapeutic regimens reflected the clinical parameters of age, comorbidity and performance status, and the biological parameters of *KRAS*, *NRAS*, and *BRAF* status; characteristics of systemic therapeutic regimens have been previously reported in details (Bruera et al. 2018; Guadagni et al. 2019). Briefly, in systemic cohort, four patients received panitumumab (6 mg/kg), one patient received panitumumab (6 mg/kg) and irinotecan (120–160 mg/m^2^), two patients received cetuximab (250 mg/m^2^) and irinotecan (120–160 mg/m^2^), one patient received cetuximab (250 mg/m^2^), irinotecan (120–160 mg/m^2^) and capecitabine (825 mg/m^2^ twice a day), two patients received aflibercpet (4 mg/kg), two patients received raltitrexed (3 mg/m^2^), two patients received regorafenib (80–160 mg), two patients received bevacizumab (5 mg/kg) and 5-fluorouracil (750–900 mg/m^2^ day), one patient received oxaliplatin (70–80 mg/m^2^) and cetuximab (250 mg/m^2^), one patient received oxaliplatin (70–80 mg/m^2^) and 5-fluorouracil (750–900 mg/m^2^ day), one patient received capecitabine (825 mg/m^2^ twice a day).

Treatment was discontinued in case of progressive disease, worsening of general conditions, severe adverse events, or patient withdrawal. Cetuximab in both cohorts and panitumumab in systemic cohort were administered according to the following conditions: EGFR overexpression; absence of mutations in *KRAS* and *NRAS* exon 2 codons 12 and 13, exon 3 codons 59 and 61 and, and exon 4 codons 117 and 146, in recurrent cancer cells or primary tumor tissues (Sepulveda et al. 2017).

### Criteria for responses and adverse events

Tumor responses were assessed in accordance with Response Evaluation Criteria in Solid Tumors (RECIST version 1.1), at 30–45 days following each loco-regional chemotherapy treatment (Eisenhauer et al. 2009). The response of patients treated prior to 2009, was re-classified retrospectively. Responses were evaluated by CT and Magnetic Resonance Imaging (MRI), and Position-emission Tomography (PET) added where applicable. Adverse events were evaluated in accordance with the Common Terminology Criteria for Adverse Events of the National Cancer Institute (CTCAE v4.03).

### Statistical analysis

Statistical analyses were performed using STATA software, version 14 (StataCorp, College Station, Texas, USA) and calculated with 95% confidence limits. Survival-rates were estimated using the Kaplan–Meier product limit estimator and no patients were lost during follow-up. Survival times were stratified according to clinical variables that may affect survival, and log-rank tests were used to assess significant differences between groups. Hazard ratios were estimated using a proportional hazard Cox regression model. Progression-free survival-time (PFS) was calculated from the initial treatment of the 3rd line. Overall survival (OS) was calculated from the initial treatment of the 3rd line to death or end of follow-up. RRC overall survival (RRC-OS) was calculated from diagnosis of RRC to death or end of follow-up.

## Results

Sixty-two patients underwent 102 HPP treatments and 55 cycles of systemic therapy. No technical, hemodynamic or vascular complications were detected during HPP procedures. There were no perfusion-related postoperative deaths and femoral cannulation was possible in all cases. Procedure-related complications and toxicities are reported in Table 3. Hematological grade 3 toxicity was identified in 8% and grade 2–3 skin toxicity detected in 24% of patients.

**Table 3:** Procedure-related complications and toxicities detected in 62 patients with URCC in progression after two lines of systemic chemotherapy and radiotherapy

	Grade	All patients (*n* = 62)	HPP/target-therapy group (*n* = 43)	Systemic therapy group (*n* = 19)
Part A: procedure-related complications				
Persistent leakage of fluid from the incision	1	2	2	0
Seroma	1	2	2	0
Wound infection	1	1	1	0
Scrotum edema	1	1	1	0
Pelvic pain	1	2	1	1
Inguinal hematoma	1	1	1	0
Port-a-cath infection	2	2	0	2
Part B: procedure-related toxicities				
Bone marrow hypocellularity	1	10	5	5
	2	0	0	0
	3	5	3	2
Platinum-induced neurotoxicity	2	5	3	2
Alopecia	2	2	2	0
Nausea and vomiting	1	6	4	2
Diarrhea	1	3	0	3
	2	4	0	4
Mucositis	3	1	0	1
Fatigue	1	5	0	5
	2	3	2	1
Skin toxicity	1	10	8	2
	2	14	8	6
	3	1	0	1

### Tumor responses

Tumor responses, considering the first two treatments for both groups, evaluated according to RECIST 1.1, are reported in Table 3. In the HPP/target-therapy group, 18/43 (41.8%) exhibited a partial response (PR), 24/43 (55.8%) stable disease (SD), and 1/43 (2.4%) exhibited disease progression (PD). The objective response rate (ORR) in this group was 41.8% and disease control rate (DCR) was 97.7%. In the systemic therapy control group, 3/19 (15.8%) exhibited PR, 5/19 (26.3%) SD, and 11/19 (57.9%) PD. The ORR and DCR in this group were 15.8 and 42.1%, respectively. The HPP/target-therapy group exhibited a significantly higher DCR compared to the systemic therapy control group (Fisher exact test, *P* = 0.001). Six of the 27 (22%) HPP-treated patients presenting with PS3, improved to PS2.

### Survival

**Fig. 2:**
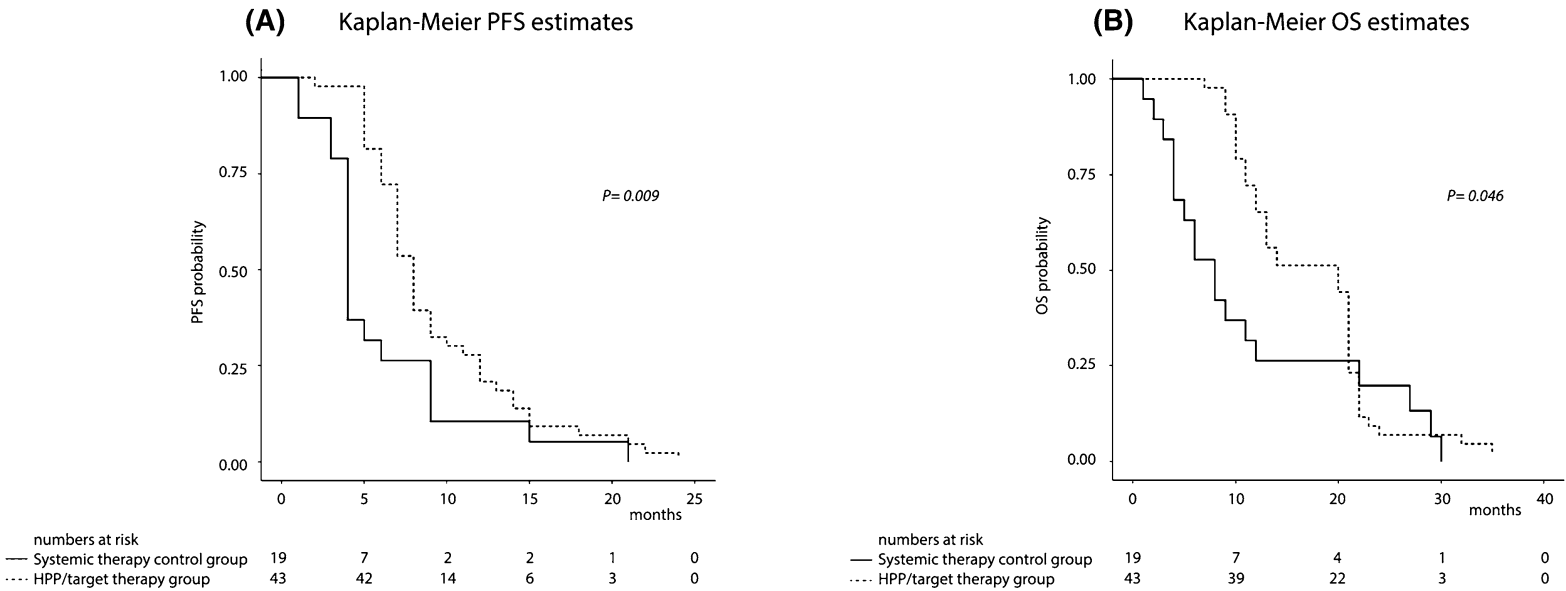
Kaplan–Meier survival estimates in 62 URRC patients from first treatment of the third line to end of follow-up: **a** progression free survival; **b** overall survival

The median follow-up time from URRC diagnosis to death or end of follow-up was 33.5 (IQR 25–40) months. By the end of follow-up, only 1 (2.3%) HPP/target therapy patient and 1 (5.3%) systemic therapy control patient were still alive. Kaplan–Meier survival analysis indicated significant differences in PFS and OS, characterized by a significant increase in median PFS (from first treatment of the third line) of 8 (IQR 6–12) months in the HPP/target therapy group compared to 4 (IQR 4–9) months in the systemic therapy control group (*P* = 0.009) (Fig. 2a), and a significant increase in median OS (from first treatment of the third line) in the HPP/target therapy group of 20 (IQR 11–21) months compared to 8 (IQR 4–17) months (*P* = 0.046) in the systemic therapy control group (Fig. 2b).

Cox univariate and multivariate analysis identified several prognostic factors for PFS and OS. Univariate analysis demonstrated that the third line treatment modality, modified Yamada’s classification (Yamada et al. 2001), other metastatic sites and ECOG PS, were significantly associated with PFS (Table 4, Part A). Of these, the third line treatment modality modified Yamada’s classification (Yamada et al. 2001), and ECOG PS were confirmed to be independent predictive factors for PFS, by multivariate analysis (Table 5, Part A). Univariate analysis (Table 4, Part B) also demonstrated that the third line treatment modality, age > 60 years, modified Yamada’s classification (Yamada et al. 2001), other metastatic sites, and ECOG PS, were associated with OS (*P* < 0.10), of which the third line treatment modality, age ≤ 60 years and modified Yamada’s classification (Yamada et al. 2001) were confirmed by multivariate analysis to be independent predictive factors for OS (Table 5, Part B).

**Table 4:** Univariate analysis, Part A: progression free survival (PFS) times from first treatment of the third line to death or last contact, in 62 URRC patients in progression after two lines systemic chemotherapy and radiotherapy

Variables (number of patients)	Median (months)/IQR	Log-Rank *χ*^2^	*P* value	HR (95% CI)	*P* value
*Part A: progression free survival*					
Third line treatment					
Systemic therapy control group (*n* = 19)	4/4–9			1.939 (1.117–3.365)	0.019
HPP/target therapy group (*n* = 43)	8/6–12	6.76	0.009		
Gender					
Male (*n* = 40)	7.5/4–10			1.230 (0.717–2.111)	0.451 (ns)
Female (*n* = 22)	7/6–10	0.67	0.412 (ns)		
Age					
≤ 60 (*n* = 29)	7/6–10			0.870 (0.521–1.452)	0.596 (ns)
> 60 (*n* = 33)	8/4–9	0.33	0.564 (ns)		
Yamada’ s modified classification (Yamada et al. 2001)					
Localized* (*n* = 13)	11/8–18				
Sacral (*n* = 37)	7/5–9			2.271 (1.126–4.580)	0.022
Lateral (*n* = 12)	5/2.5–6	21.92	0.001	6.675 (2.679–16.629)	0.001
Other sites of metastases					
Yes (*n* = 37)	7/4–9			1.645 (0.964–2.806)	0.068 (ns)
Not (*n* = 25)	8/5–14	4.00	0.045		
ECOG					
1 (*n* = 10)	14.5/9–21				
2 (*n* = 12)	8.5/6.5–12.5				
3 (*n* = 40)	6/4–8	15.86	0.001	1.931 (1.342–2.778)	0.001
Target-therapy					
Yes (*n* = 38)	7.5/5–12			1.198 (0.708–2.025)	0.500 (ns)
Not (*n* = 24)	6.5/5–8.5	0.53	0.464 (ns)		
*Part B: survival*					
Third line treatment					
Systemic therapy control group (*n* = 19)	8/4–17			1.703 (0.965–3.005)	0.066 (ns)
HPP/target therapy group (*n* = 43)	20/11–21	3.98	0.046		
Gender					
Male (*n* = 40)	13/8.5–21			1.346 (0.785–2.307)	0.280 (ns)
Female (*n* = 22)	15/11–22	1.35	0.245		
Age					
≤ 60 (*n* = 29)	12/10–20			1.953 (1.125–3.389)	0.017
> 60 (*n* = 33)	20/9–22	6.76	0.009		
Yamada’ s modified classification (Yamada et al. 2001)					
Localized* (*n* = 13)	22/21–29				
Sacral (*n* = 37)	12/9–21			2.382 (1.218–4.655)	0.011
Lateral (*n* = 12)	11.5/5–13	15.34	0.001	4.738 (1.993–11.262)	0.001
Other sites of metastases					
Yes (*n* = 37)	12/8–20			1.635 (0.963–2.774)	0.068 (ns)
Not (*n* = 25)	21/11–22	3.89	0.048		
ECOG					
1 (*n* = 10)	21/9–32				
2 (*n* = 12)	21.5/17–25				
3 (*n* = 40)	11/8.5–18.5	16.19	0.001	2.127 (1.372–3.298)	0.001
Target-therapy					
Yes (*n* = 38)	14/10–21				
Not (*n* = 24)	11.5/9–21	0.01	0.913	1.023 (0.604–1.748	0.919 (ns)

Patients were stratified according to third-line treatment modality, gender, age, Yamada’s modified classification, other metastatic sites, and ECOG. Part B: survival times

* Including also cases with invasion of uterus, vagina, bladder, prostate, and seminal vesicles

**Table 5:** Multivariate analysis, Part A: progression free survival (PFS) variables. Part B: survival (OS) variables

Variables (number of patients)	HR (95% CI)	*P* value
*Part A: progression free survival*		
Third line treatment		
Systemic therapy control group (*n* = 19)	2.182 (1.132–4.204)	0.020
HPP/target therapy group (*n* = 43)		
Yamada’ s modified classification (Yamada et al. 2001)		
Localized* (*n* = 13)		
Sacral (*n* = 37)	2.569 (1.061–6.222)	0.036
Lateral (*n* = 12)	8.703 (2.535–29.872)	0.001
Other sites of metastases		
Yes (*n* = 37)	0.783 (0.398–1.542)	0.481 (ns)
Not (*n* = 25)		
ECOG		
1 (*n* = 10)		
2 (*n* = 12)		
3 (*n* = 40)	1.492 (1.003–2.220)	0.048
*Part B: survival*		
Third line treatment		
Systemic therapy control group (*n* = 19)	4.393 (1.992–9.684)	0.001
HPP/target therapy group (*n* = 43)		
Age		
≤ 60 (*n* = 29)	2.360 (1.317–4.229)	0.004
> 60 (*n* = 33)		
Yamada’ s modified classification (Yamada et al. 2001)		
Localized* (*n* = 13)		
Sacral (*n* = 37)	5.306 (1.861–15.126)	0.002
Lateral (*n* = 12)	11.086 (2.847–43.155)	0.001
Other sites of metastases		
Yes (*n* = 37)	1.487 (0.916–2.413)	0.108 (ns)
Not (*n* = 25)		
ECOG		
1 (*n* = 10)		
2 (*n* = 12)		
3 (*n* = 40)	0.827 (0.417–1.636)	0.586 (ns)

* Including also cases with invasion of uterus, vagina, bladder, prostate, and seminal vesicles

In this 62-patient cohort, the median OS from RRC diagnosis to death or end of follow-up (RRC-OS) was 33.5 (IQR 25–40) months. No one in the HPP/target therapy group underwent further treatment, whereas 6 (31.5%) patients in the systemic treatment control group underwent further lines of treatment.

## Discussion

URRC management requires a multidisciplinary approach, involving standard systemic chemotherapy, radiotherapy and targeted therapy, with locoregional HPP considered an addition to standard approaches or as an alternative palliative option for patients unsuitable for systemic therapy. In the current report on URRC patients, we provide the first comparative evaluation, in terms of safety and efficacy, of locoregional HPP/target therapy, based upon precision oncotherapy assays of fluid biopsies, to traditional third-line systemic therapy, defined according to clinical parameters of age, comorbidity and performance status, and biological parameters of *KRAS*, *NRAS*, and *BRAF* status.

Previously, we reported an OS of 4 months in URRC patients progressing after second-line treatments (Bruera et al. 2014), consistant with a recently reported ≈ 5 months median OS for the placebo arm of phase III trials, utilising innovative regorafenib and TAS-102 third-line treatments (Li et al. 2015; Mayer et al. 2015). These studies demonstrated an approximate PFS of 2 months and an approximate OS of 7 months for regorafenib and TAS-102, with a safety profile characterized by hand-foot skin reactions and fatigue for regorafenib, and myelosuppression for TAS-102 (Li et al. 2015; Mayer et al. 2015).

The present study indicates that the HPP/target-therapy group DCR, with drug regimens selected by precision oncotherapy liquid biopsy, is potentially superior (*P* = 0.001) than that of systemic therapy, suggesting that regimens that combine locoregional and systemic therapy may offer better short-term control of URRC. Efficacy analyses also resulted in a significantly higher median PFS (from the first third-line treatment) for HPP/target-therapy of 8 months compared to 4 months in systemic therapy controls (*P *= 0.009), and the median OS (from the first third-line treatment) of 20 months for the HPP/target-therapy group was also significantly higher than that of 8 months for systemic therapy controls (*P* = 0.046), further indicating that URRC patients may derive significant benefit from combining locoregional and systemic therapy.

Currently, precision oncotherapy and chemo-sensitivity assays are performed on tissue specimens with less invasive liquid biopsies only recently considered a viable alternative (Yoon and Kim 2014; Sepulveda et al. 2017; Karachaliou et al. 2015). The methods employed in this study for preserving and transporting CTC-containing blood samples are in line with recent reports (Qin et al. 2014; Kang et al. 2016), and the PCR-based techniques used for precision oncotherapy are in accordance with recent guidelines for colorectal cancer treatments (Kentaro et al. 2018).

It was somewhat surprising that CTCs from 79% of patient exhibited moderate to high sensitivity to MMC, compared to 7% for irinotecan, 30% for oxaliplatin, 2.3% for 5-fluorouracil, 14% for raltitrexed, 4.6% for alkeran, and 4.6% for carboplatin, considering that MMC is currently recognized to be not very active against colorectal cancer cells. As concerning MMC administration, in a previous published paper (Guadagni et al. 2017) we demonstrated that the MMC maximum concentration (Cmax) in the isolated pelvic compartment of 18 patients was approximately 60 μg/ml, 100 times higher than the 2 μM concentration to which CTCs were exposed during in vitro chemosensitivity tests. Moreover, in vitro CTCs MMC chemosensitivity tests were not performed under hypoxia, and it has been demonstrated that MMC is 10 times more cytotoxic in hypoxic conditions (Teicher et al. 1981) as administered during HPP procedures. These sensitivity differences may be explained either by previous systemic chemotherapy for metastatic disease or by observations that CTCs from 35% of patients exhibited significant ERCC1 and GST over-expression (> 10%), involved in resistance to platinum compounds (Yu et al. 2009), CTCs from 34.8% of patients showed ≥ 5% TYMS, or DHFR, or SHMT1 expression, involved in resistance to 5-fluorouracil (Jensen et al. 2008; Di Paolo and Chu 2004), and CTCs from 77% of patients showed very high multi-drug resistance gene (*MDR1*) overexpression (≥ 65%). This illustrates the need for more dynamic evaluation of tumor samples during metastatic progression, a strong advocate for the use of less-invasive liquid biopsies that more accurately assess the current mutational status of tumors, as a pre-requisite for more accurate selection of a personalized therapeutic strategy.

Safety profiling detected G2–G3 hematological toxicity in 7% of the HPP/target therapy group and 10% of the systemic therapy group, G2–G3 skin toxicity in 19% of the HPP/target therapy group and 37% of the systemic therapy group, and multivariate analysis demonstrated that the combination of locoregional and systemic treatment, age > 60 years, and localized recurrence, represent independent predictors of prolonged OS.

Limitations of this study are: (i) small sample size in both cohorts; (ii) the study is retrospective and not prospective; (iii) single-center study; (iv) selection bias according to high percentage of other metastatic sites in both patients cohorts; (v) risk of selection bias because control group patients were treated with systemic therapy only and without locoregional chemotherapy; (vi) treatment bias in the experimental cohort and in the control cohort as consequence of inhomogeneous drugs regimens suggested by precision oncotherapy; (vii) the results in the experimental group could be overstated and in general the results of the study are not conclusive.

In conclusion and in spite of the limitations of this study, the results in terms of safety, tolerability and prolonged local control of locoregional chemotherapy (HPP) with target-therapy drug regimens selected by liquid biopsy precision oncotherapy as third-line treatment of non-responsive URRC patients, deserve, in our opinion, further evaluation in a future prospective randomized trial as alternative to third-line systemic therapy only.
